# Stabilizing the Inverted Phase of a WSe_2_/BLG/WSe_2_ Heterostructure via Hydrostatic Pressure

**DOI:** 10.1021/acs.nanolett.3c03029

**Published:** 2023-10-16

**Authors:** Máté Kedves, Bálint Szentpéteri, Albin Márffy, Endre Tóvári, Nikos Papadopoulos, Prasanna K. Rout, Kenji Watanabe, Takashi Taniguchi, Srijit Goswami, Szabolcs Csonka, Péter Makk

**Affiliations:** † Department of Physics, Institute of Physics, Budapest University of Technology and Economics, Műegyetem rkp. 3, Budapest H-1111, Hungary; ‡ MTA-BME Correlated van der Waals Structures Momentum Research Group, Műegyetem rkp. 3, Budapest H-1111, Hungary; ¶ MTA-BME Superconducting Nanoelectronics Momentum Research Group, Műegyetem rkp. 3, H-1111 Budapest, Hungary; § QuTech and Kavli Institute of Nanoscience, Delft University of Technology, Delft 2600 GA, The Netherlands; ∥ Research Center for Functional Materials, National Institute for Materials Science, 1-1 Namiki, Tsukuba 305-0044, Japan; ⊥ International Center for Materials Nanoarchitectonics, National Institute for Materials Science, 1-1 Namiki, Tsukuba 305-0044, Japan

**Keywords:** bilayer graphene, WSe_2_, spin−orbit interaction, band inversion, pressure, transport measurements

## Abstract

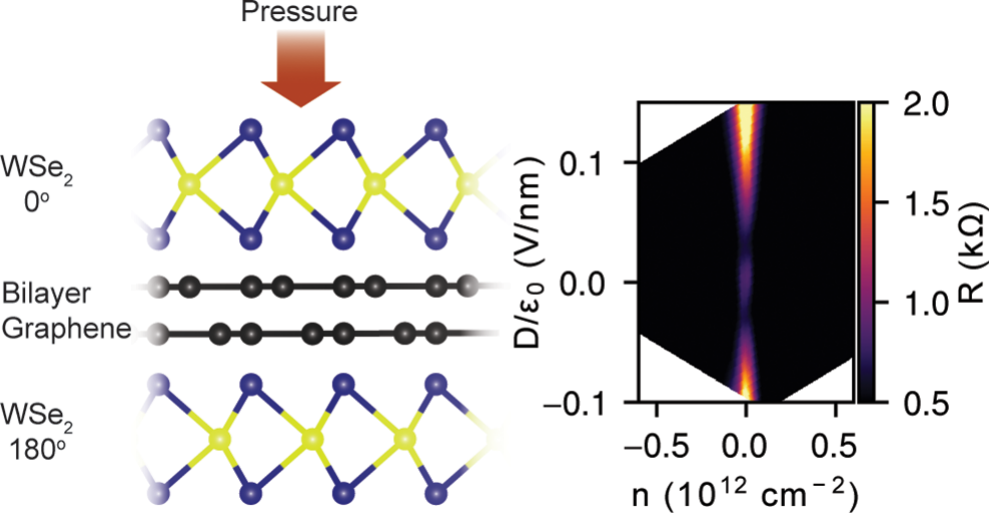

Bilayer graphene (BLG) was recently shown to host a band-inverted phase with unconventional topology emerging from the Ising-type spin–orbit interaction (SOI) induced by the proximity of transition metal dichalcogenides with large intrinsic SOI. Here, we report the stabilization of this band-inverted phase in BLG symmetrically encapsulated in tungsten diselenide (WSe_2_) via hydrostatic pressure. Our observations from low temperature transport measurements are consistent with a single particle model with induced Ising SOI of opposite sign on the two graphene layers. To confirm the strengthening of the inverted phase, we present thermal activation measurements and show that the SOI-induced band gap increases by more than 100% due to the applied pressure. Finally, the investigation of Landau level spectra reveals the dependence of the level-crossings on the applied magnetic field, which further confirms the enhancement of SOI with pressure.

Van der Waals (VdW) engineering provides a powerful method to realize electronic devices with novel functionalities via the combination of multiple 2D materials.^1^ An exciting example is the case of graphene connected to materials with large intrinsic spin–orbit interaction (SOI), which allows the generation of an enhanced SOI in graphene via proximity effect.^2−26^ This, on the one hand, is compelling in the case of spintronics devices since the large spin diffusion length in graphene heterostructures^27−29^ could be complemented with electrical tunability^30−32^ or charge-to-spin conversion effects.^33^ Moreover, it is also interesting from a fundamental point of view since graphene with intrinsic SOI was predicted to be a topological insulator.^34^ The observation of increased SOI was demonstrated in the past few years in both single layer^12−20^ and recently in bilayer graphene (BLG).^14,21−26^ It was found that one of the dominating spin–orbit terms is the Ising-type valley-Zeeman term which is an effective magnetic field acting oppositely in the two valleys, and could enable such exciting applications as a valley-spin valve in BLG.^35^ Recent compressibility measurements^21^ have shown that BLG encapsulated in tungsten-diselenide (WSe_2_) from both sides hosts a band-inverted phase if the sign of induced SOI is different for the two WSe_2_ layers. In practice, this can be achieved if the twist angle between the two WSe_2_ layers is, for example, 180°.^7,11,36^

In this article, we experimentally investigate the SOI induced in BLG symmetrically encapsulated in WSe_2_ (WSe_2_/BLG/WSe_2_) via transport measurements. We present resistance measurements as a function of charge carrier density (*n*) and the transverse displacement field (*D*) at ambient pressure and demonstrate the appearance of the inverted phase (IP). In order to stabilize this phase, we employ our recently developed setup^37,38^ to apply a hydrostatic pressure (*p*), which allows us to decrease the distance between the WSe_2_ layers and bilayer graphene and to boost the SOI as we have recently demonstrated on single layer graphene.^39^ The sample is placed in a piston–cylinder pressure cell, where kerosene acts as the pressure mediating medium. More details about this can also be found in Methods. To confirm the increased SOI, we present thermal activation measurements where the evolution of the SOI-induced band gap can be estimated as a function of *D* and *p*. Finally, we further investigate the induced SOI with quantum Hall measurements by tracking the Landau level crossings as a function of the magnetic field.

To reveal the band-inverted phase arising from the Ising SOI in BLG, we show the low-energy band structure of WSe_2_/BLG/WSe_2_ in Figure 1, calculated using a continuum model by following in the footsteps of ref.^7^ The effect of the WSe_2_ layers in the proximity of BLG can be described by the Ising SOI terms λ_I_^t^ and λ_I_^b^ that couple only to the top or bottom layer of BLG and act as a valley-dependent effective magnetic field. For WSe_2_ layers rotated with respect to each other with 180°, the induced SOI couplings will have opposite sign.^7,11,36^ This is taken into account by the opposite signs of λ_I_^t^ and λ_I_^b^. The transverse displacement field (*D*) in our measurements can be modeled by introducing an interlayer potential difference , where *e* is the elementary charge, ϵ_0_ is the vacuum permittivity, *d* = 3.3 Å is the separation of BLG layers, and ϵ_BLG_ is the effective out-of-plane dielectric constant of BLG.

**Figure 1:**
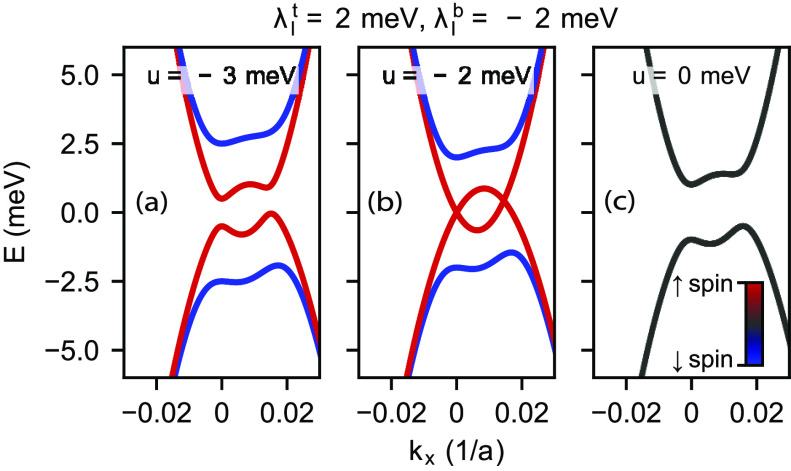
(a–c) Calculated band structure around the **K**-point for different values of the interlayer potential difference *u*. Color scale corresponds to the spin polarization of the bands.

Figure 1a–c shows the calculated band structure around the **K**-point for different values of *u*, using the parameter values λ_I_^t^ = −λ_I_^b^ = 2 meV. Details of the modeling can be found in the Supporting Information. First of all, for |*u*| > |λ_I_^t^| = |λ_I_^b^|, we can see the opening of a band gap (Figure 1a), as expected for BLG in a transverse displacement field.^40,41^ On the other hand, as opposed to pristine BLG, the bands are spin-split, and the direction of this spin splitting is opposite for the valence and conduction bands. This is a direct consequence of the opposite sign of λ_I_^t^ and λ_I_^b^ as the valence and conduction bands are localized on different layers due to the large *u*. The band structure in the **K′**-valley is similar except that the spin-splitting is reversed due to time reversal symmetry. For |*u*| = |λ_I_^t,b^| (Figure 1b), the *u*-induced band gap approximately equals the spin splitting induced by the Ising SOI and the bands touch. Finally, for |*u*| < |λ_I_^t,b^| (Figure 1c), a band gap reopens and we observe spin-degenerate bands for *u* = 0, separated by a gap comparable in size to the Ising SOI terms (Δ ≈ |λ_I_^t^ – λ_I_^b^|/2). This gapped phase is distinct from the band insulating phase at large *u* in that the valence and conduction bands are no longer layer polarized, hence it is usually referred to as inverted phase (IP). It is worth mentioning that the IP at |*u*| < |λ_I_^t^| is weakly topological unlike the trivial band insulating phase.^42,43^

Our device consists of a BLG flake encapsulated in WSe_2_ and hexagonal boron nitride (hBN) on both sides, as illustrated in Figure 2a. To enable transport measurements, we fabricated NbTiN edge contacts in a Hall bar geometry. The device also features a graphite bottomgate and a metallic topgate that allow the independent tuning of *n* and *D*. See the Supporting Information for more details about sample fabrication and geometry. The results on similar devices with very similar findings are also shown in the Supporting Information.

**Figure 2:**
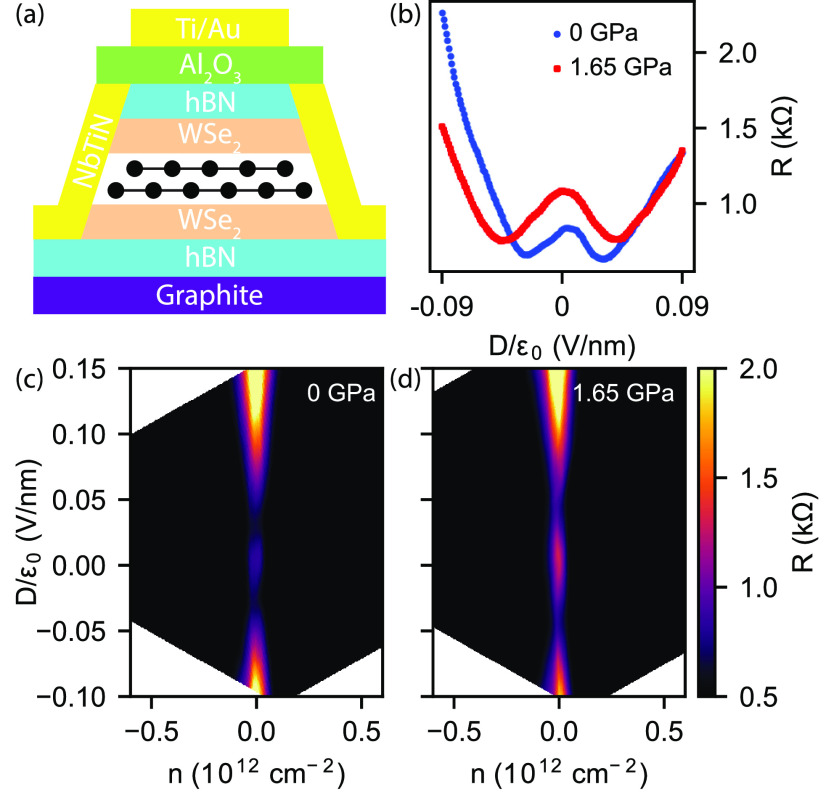
(a) Schematic representation of the measured device. Bilayer graphene is symmetrically encapsulated in WSe_2_ and hBN. (b) Line trace of the four-terminal resistance along the CNL for ambient pressure (blue) and *p* = 1.65 GPa (red). (c, d) Four-terminal resistance map as a function of charge carrier density *n* and displacement field *D* measured at (c) ambient pressure and (d) an applied pressure of 1.65 GPa. The alternating low and high resistance regions along the CNL indicate the closing and reopening of a band gap in the bilayer graphene.

Figure 2c shows the resistance measured in a four-terminal geometry as a function of *n* and *D* at ambient pressure at 1.4 K temperature. As expected for BLG, we observe the opening of a band gap at large displacement fields along the charge neutrality line (CNL) at *n* = 0, indicated by an increase of resistance. In accordance with the theoretical model and previous compressibility measurements,^21^ we also observe two local minima separated by a resistance peak at *D* = 0 in agreement with the closing and reopening of the band gap signaling the transition between the band insulator and the IP. This observation is further emphasized in Figure 2b, where a line trace (blue) of the resistance is shown as a function of *D*, measured along the CNL. It is important to note that during the fabrication process the rotation of WSe_2_ layers was not controlled. However, from theoretical predictions,^7,11,36^ we only expect to observe signatures of the IP for a suitable range of rotation angles between the two WSe_2_ layers (e.g., ∼180°). This is further supported by the fact that not all devices fabricated showed the IP. An example for this case is shown in the Supporting Information, where only the band insulating regime can be observed in the resistance map.

To boost the induced SOI and stabilize the IP, we applied a hydrostatic pressure of *p* = 1.65 GPa and repeated the previous measurement. Figure 2d shows the *n*–*D* map of the resistance after applying the pressure. Although the basic features of the resistance map are similar, two consequences of applying the pressure are clearly visible. First, as also illustrated in Figure 2b, the peak resistance in the IP at *D* = 0 increased by ∼25%. Second, the displacement field required to close the gap of the IP increased significantly, by about 70%. Both of these observations can be accounted for by an increase in the Ising SOI term that results in a larger gap at *D* = 0 and subsequently in a larger displacement field needed to close the gap. Although the shift of resistance minima could be explained by the increase of ϵ_BLG_ or the decrease of interlayer separation *d*, these altogether are not expected to have greater effect than ∼20%.^44,45^ It is also worth mentioning that the lever arms also change due to the applied pressure, changing the conversion from gate voltages to *n* and *D*; however, we have corrected for this effect by experimentally determining them from quantum Hall measurements (see the Supporting Information).

To quantify the increase in the SOI gap due to hydrostatic pressure, we performed thermal activation measurements along the CNL for several values of *D*. Figure 3a demonstrates the evolution of resistance as a function of 1/*T* for selected values of *D* at ambient pressure. From this, we extract the band gap using a fit to the high-temperature, linear part of the data where thermal activation–ln(*R*) ∝ Δ/2*k*_B_*T* – dominates over hopping-related effects.^46^Figure 3b shows the extracted gap values as functions of *D* with and without applied pressure. First of all, a factor of 2 increase is clearly visible in the gap at *D* = 0 for *p* = 1.65 GPa, which is consistent with the observed increase of resistance. Second, the higher *D* needed to reach the gap minima is also confirmed. We also note that the band gap cannot be fully closed which we attribute to spatial inhomogeneity in the sample.

**Figure 3:**
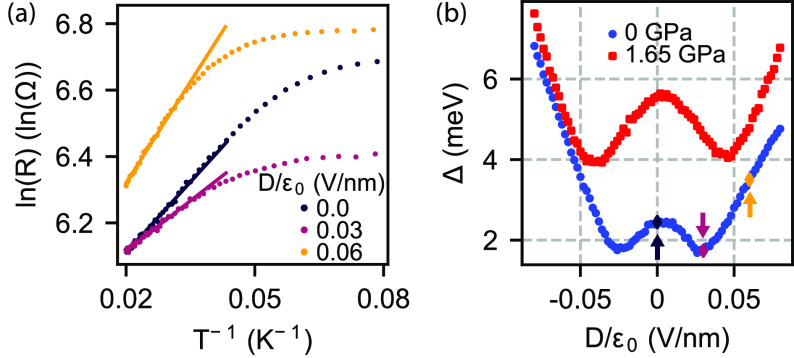
Thermal activation measurements along the charge neutrality line. (a) Arrhenius plot of the resistance at ambient pressure for selected values of *D*. Solid lines are fits to the linear parts of the data from which the band gap values were obtained. (b) Gap Δ as a function of displacement field at ambient pressure (blue) and an applied pressure of 1.65 GPa. Arrows indicate the *D* values for which the activation data are shown in a.

The experimentally determined band gaps allow us to quantify the SOI parameters. By adjusting the theoretical model to match the positions of the gap minima and the opening of the trivial gap for *p* = 0, we extract λ_I_^t^ = −λ_I_^b^ = 2.2 ± 0.4 meV. Similarly, we can extract the SOI parameters at *p* = 1.65 GPa. For these, we obtain λ_I_^t^ = −λ_I_^b^ = 5.6 ± 0.6 meV. The SOI parameters extracted from the minima give the same order of magnitude estimate as the gaps at *D* = 0 extracted from thermal activation directly. A more detailed discussion of the extraction and possible errors is given in the Supporting Information. We expect that all layer distances (e.g., hBN-hBN, BLG-WSe_2_, and *d*) change due to the applied pressure as it is also reflected in the change of lever arms. The extracted increase of SOI strength due to the change of BLG-WSe_2_ distances is consistent with theoretical predictions in ref (37), where almost a factor of 3 increase was predicted for an applied pressure of 1.8 GPa. Importantly, we have found similar results in two further devices shown in the Supporting Information.

The quantum Hall effect in BLG provides us another tool to investigate the Ising SOI induced by the WSe_2_ layers. The 2-fold degeneracy of valley isospin (ξ = +, – ), the first two orbitals (*N* = 0, 1) and spin (σ = ↑, ↓) give rise to an 8-fold degenerate Landau level (LL) near zero-energy.^47−49^ This degeneracy is weakly lifted by the interlayer potential difference, Zeeman energy, coupling elements between the BLG layers^50^ and the induced Ising SOI.^22^ We can obtain the energy spectrum of this set of eight closely spaced sublevels, labeled by |ξ, *N*, σ⟩, by introducing a perpendicular magnetic field in our continuum model, as detailed in.^50^ This is shown in Figure 4a for *B* = 8.5 T as a function of the interlayer potential (*u*). LLs with different ξ reside on different layers of the BLG, and therefore *u* induces a splitting between these levels. Second, the finite magnetic field causes the Zeeman-splitting of levels with different σ. Finally, the Ising SOI induces an additional effective Zeeman field associated with a given layer, further splitting the levels. The key feature that should be noted here is that for a given filling factor ν, crossings of LLs can be observed and the position of crossing points along the *u* axis depend on SOI parameters as well as on the magnetic field. These level crossings manifest as sudden changes of resistance in our transport measurements as is illustrated in Figure 4b. Here, the *n*–*D* map of the resistance is shown as measured at *B* = 8.5 T with fully developed resistance plateaus (due to the unconventional geometry, see the Supporting Information) corresponding to the sublevels of ν ∈ [−4,4]. For a given filling factor ν, we observe 4 – |ν| different *D* values where the resistance deviates from the surrounding plateau corresponding to the crossing of LLs, as expected from the model.

**Figure 4:**
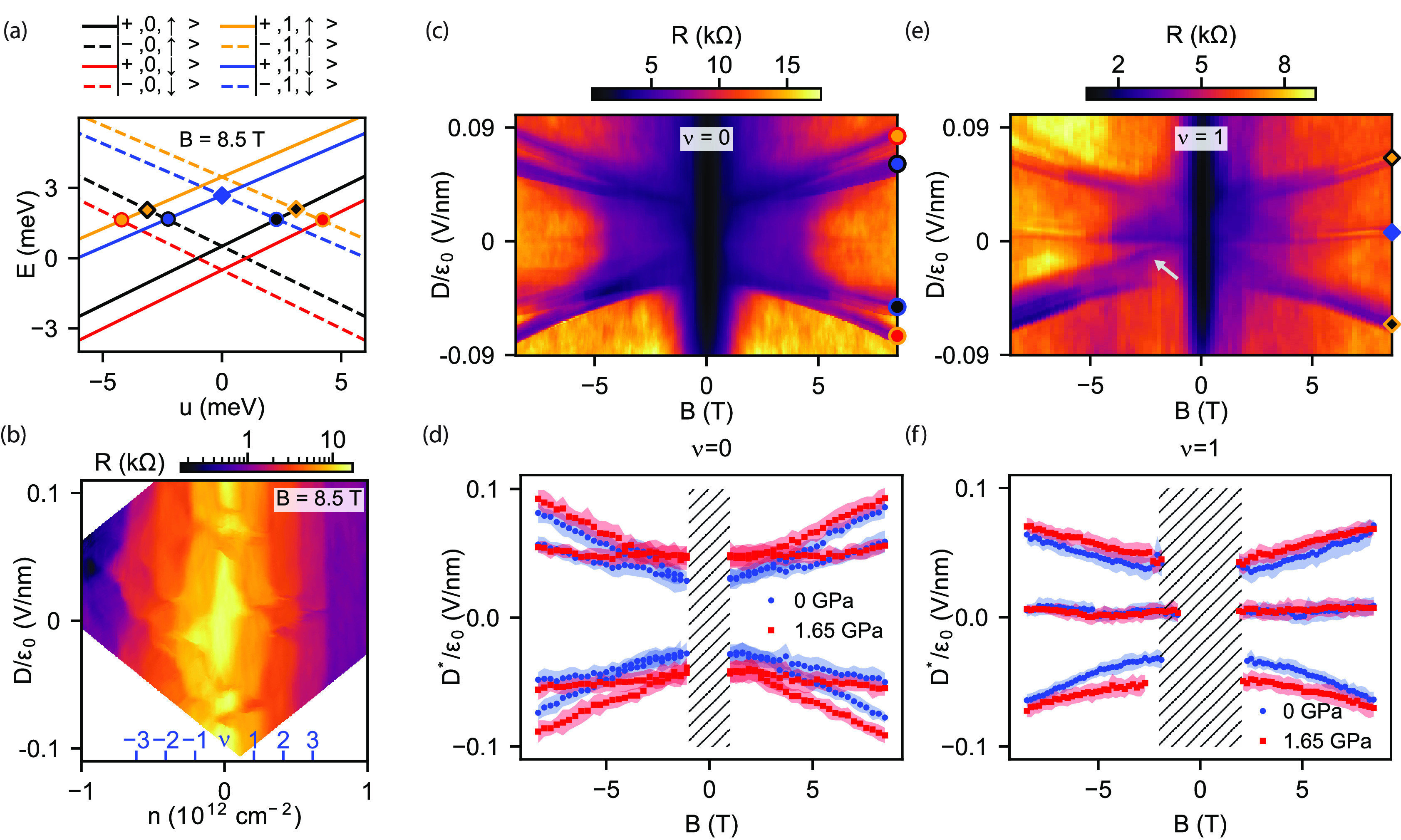
(a) Low energy Landau level spectrum at *B* = 8.5 T obtained from single-particle continuum model with λ_I_^t^ = −λ_I_^b^ = 2 meV. (b) Four-terminal resistance as a function of *n* and *D* measured at *B* = 8.5 T out-of-plane magnetic field and ambient pressure. Resistance plateaus correspond to different ν filling factors. Abrupt changes in resistance at a given ν value as a function of *D* indicate the crossings of LLs. (c, e) Measurements of LL crossings as a function of *B* for ν = 0 and ν = 1, respectively, for *p* = 0. Symbols denote LL crossings shown in a. (d, f) Critical displacement field D* corresponding to LL crossings for ν = 0 and ν = 1 extracted from *D* – *B* maps measured at *p* = 0 (blue, see c, e) and *p* = 1.65 GPa (red).

The evolution of LL crossings with *B* can be observed by performing measurements at fixed filling factors, as shown in Figure 4c and e for ν = 0 and ν = 1, respectively. During the latter measurement, carrier density *n* was tuned such that the filling factor given by ν = *nh*/*eB* was kept constant. On both panels, we can observe 4 – ν LL crossings that evolve as *B* is tuned, until they disappear at low magnetic fields where we can no longer resolve LL plateaus. This *B*-dependent behavior enables us to investigate the effect of SOI on the LL structure. Figure 4d and f shows the critical displacement field D* values, where LL crossings can be observed, extracted from Figure 4c and e and similar maps measured at *p* = 1.65 GPa (see the Supporting Information). For ν = 0 (Figure 4d), the most important observation is that the crossing points do not extrapolate to zero as *B* → 0 T, which is a direct consequence of the induced Ising SOI. It is also clearly visible that due to the applied pressure, |D*| is generally increased, especially at lower *B*-fields, indicating that the Ising SOI has increased, in agreement with our thermal activation measurements. For ν = 1 (Figure 4f), similar trends can be observed. The two LL crossings at finite *D* saturate for small *B*, while the third crossing remains at *D* = 0. We note that the D*(*B*) curves for *p* = 1.65 GPa cannot be scaled down to the *p* = 0 curves, which confirms that our observations cannot simply be explained by an increased ϵ_BLG_ or decreased interlayer separation distance, but are the results of enhanced SOI. We also point out that some lines which extrapolate to *D* = 0 can also be observed (e.g., Figure 4e, gray arrow). This could also be explained by sample inhomogeneity. It is also important to note that our single-particle model fails to quantitatively predict the *B*-dependence of the LL crossings indicating the importance of electron–electron interactions (see the Supporting Information).

In conclusion, we showed that the IP observed in BLG symmetrically encapsulated between twisted WSe_2_ layers can be stabilized by applying hydrostatic pressure, which enhances the proximity induced SOI. We presented thermal activation measurements as a means to quantify the Ising SOI parameters in this system and showed an increase of approximately 150% due to the applied pressure. In order to gain more information on the twist angle dependence of the SOI, a more systematic study with several samples with well-controlled twist angles is needed. The enhancement of Ising SOI with pressure was further confirmed from quantum Hall measurements. However, to extract SOI strengths from these measurements, more complex models are needed that also take into account interaction effects. Our study shows that the hydrostatic pressure is an efficient tuning knob to control the induced Ising SOI and thereby the topological phase in WSe_2_/BLG/WSe_2_.

The IP has a distinct topology from the band insulator phase at large *D*, and the presence of edge states are expected.^42^ The presence of these states should be studied in better defined sample geometries^51,52^ or using supercurrent interferometry.^53,54^ Opposed to the weak protection of the edge states in this system, a strong topological insulator phase is predicted in ABC trilayer graphene.^43,55^ Furthermore, pressure could also be used in case of magic-angle twisted BLG, in which topological phase transitions between different Chern insulator states are expected as a function of SOI strength.^56^

## Methods

### Sample Fabrication

The dry-transfer technique with PC/PDMS hemispheres is employed to stack hBN (35 nm)/WSe_2_ (19 nm)/BLG/WSe_2_ (19 nm)/hBN (60 nm)/graphite. To fabricate electrical contacts to the Hall bar, we use e-beam lithography patterning followed by a reactive ion etching step using CHF_3_/O_2_ mixture and finally deposit Ti (5 nm)/NbTiN (100 nm) by dc sputtering. We deposit Al_2_O_3_ (30 nm) using ALD which acts as the gate dielectric and isolates the ohmic contacts from the top gate. Finally, the top gate is defined by e-beam lithography and deposition of Ti (5 nm)/Au (100 nm).

### Transport Measurements

Transport measurements were carried out in an Oxford cryostat equipped with a variable temperature insert (VTI) at a base temperature of 1.4 K (unless otherwise stated). Measurements were performed using the lock-in technique at 1.17 kHz frequency.

### Pressurization

The sample is first bonded to a high pressure sample holder and placed in a piston–cylinder pressure cell, where kerosene acts as the pressure mediating medium. To change the applied pressure, the sample is warmed up to room temperature where the pressure is applied using a hydraulic press and the sample is cooled down again. Our pressure cell is described in more detail in ref.^39^

## Data Availability

Source data of the measurements and the Python code for the simulation are publicly available at 10.5281/zenodo.8406628.

## Supporting Information Available

Details of device fabrication, optical image of the device, measurement setup, *n*–*D* conversion, determination of lever arms, details of band structure calculation, extended activation data, details of the method to extract SOI strength, additional quantum Hall measurement data, and measurements on two-terminal devices (PDF)
