# Supplementary material for: Stabilizing the Inverted Phase of a WSe2/BLG/WSe2 Heterostructure via Hydrostatic Pressure
Source: Nano Lett. 2023 Oct 16;23(20):9508–14. doi: 10.1021/acs.nanolett.3c03029 (PMC10603803; doi:10.1021/acs.nanolett.3c03029)
Supplement: Supplementary file 1 — nl3c03029_si_001.pdf [file nl3c03029_si_001.pdf]

Supporting Information: Stabilizing the inverted phase of a WSe_2/BLG/WSe_2 heterostructure via hydrostatic pressure

Máté Kedves,[†,‡] Bálint Szentpéteri,[†,‡] Albin Márffy,[†,¶] Endre Tóvári,[†,‡] Nikos Papadopoulos,[§] Prasanna K. Rout,[§] Kenji Watanabe,[‖] Takashi Taniguchi,[⊥] Srijit Goswami,[§] Szabolcs Csonka,[†,¶] and Péter Makk[*,†,‡]

†*Department of Physics, Institute of Physics, Budapest University of Technology and Economics, Műegyetem rkp. 3., H-1111 Budapest, Hungary*

‡*MTA-BME Correlated van der Waals Structures Momentum Research Group, Műegyetem rkp. 3., H-1111 Budapest, Hungary*

¶*MTA-BME Superconducting Nanoelectronics Momentum Research Group, Műegyetem rkp. 3., H-1111 Budapest, Hungary*

§*QuTech and Kavli Institute of Nanoscience, Delft University of Technology, 2600 GA Delft, The Netherlands*

‖*Research Center for Functional Materials, National Institute for Materials Science, 1-1 Namiki, Tsukuba 305-0044, Japan*

⊥*International Center for Materials Nanoarchitectonics, National Institute for Materials Science, 1-1 Namiki, Tsukuba 305-0044, Japan*

E-mail: makk.peter@ttk.bme.hu

S1 Device geometry and measurement setup

The measured sample is shown in Fig. S1. The dry-transfer technique with PC/PDMS hemispheres is employed to stack hBN (35 nm)/WSe_2 (19 nm)/BLG/WSe_2 (19 nm)/hBN (60 nm)/graphite. To fabricate electrical contacts to the Hall bar, we use e-beam lithography patterning followed by a reactive ion etching step using CHF_3/O_2 mixture and finally deposit Ti (5nm)/NbTiN (100 nm) by dc sputtering. We deposit Al_2O_3 (30 nm) using ALD which acts as the gate dielectric. Finally, the top gate is defined by e-beam lithography and deposition of Ti (5 nm)/Au (100 nm). We note that the heterostructure was not etched into a Hall-bar shape after the contacts were deposited. Therefore current can flow along the pristine edges of the BLG layer. For the quantum Hall measurements, this results in a nontrivial sample geometry which could result in the mixing of longitudinal and transverse resistances. During the fabrication process, the alignment of WSe_2 layers was not controlled.

Transport measurements were carried out in an Oxford cryostat equipped with a variable temperature insert (VTI) at a base temperature of 1.4 K (unless otherwise stated). Measurements were performed using lock-in technique with 0.1 mV AC voltage excitation at 1171 Hz frequency. Measurements presented in the main text were conducted on device S1: the AC voltage bias was applied between contacts A and D while the four-terminal voltages were measured between B and C.

To apply hydrostatic pressure, the sample is first bonded to a high pressure sample holder and placed in a piston-cylinder pressure cell as detailed in Ref. [S1] where kerosene acts as the pressure mediating medium. To change the applied pressure the sample is warmed up to room temperature where the pressure is applied using a hydraulic press and the sample is cooled down again.

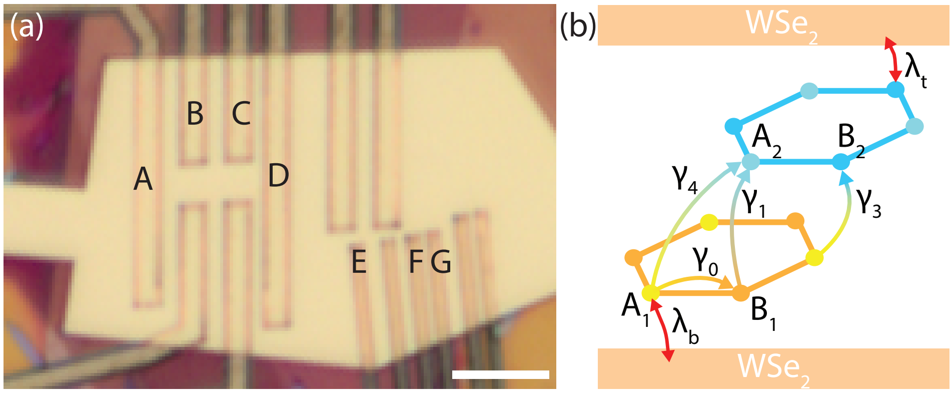

Figure S1: (a) Optical image of the measure device. The scale bar is 4 μm. (b) Schematic illustration of the heterostructure, showing the hopping terms in the BLG and the layer dependent SOC terms, induced by the WSe_2 layers.

S2 Continuum model of WSe2/BLG/WSe2

S2.1 Low-energy Hamiltonian

We model the studied heterostructure with the low-energy Hamiltonian of bilayer graphene with an additional spin–orbit coupling term, which is different on the two graphene layers, induced by the proximity of the two WSe_2 layers. In the basis of the 4 atom unit cell,

$(|C_{\rm A1}\rangle, |C_{\rm B1}\rangle, |C_{\rm A2}\rangle, |C_{\rm B2}\rangle) \otimes (|\uparrow\rangle, |\downarrow\rangle)$ the Hamiltonian is written as

$$\mathcal{H} = \mathcal{H}_{\rm BLG} + \mathcal{H}_{\rm SOC}, \tag{S1}$$

$$\mathcal{H}_{\rm BLG} = \begin{pmatrix} u/2 & v_0\pi^\dagger & -v_4\pi^\dagger & v_3\pi \\ v_0\pi & u/2+\Delta' & \gamma_1 & -v_4\pi^\dagger \\ -v_4\pi & \gamma_1 & -u/2+\Delta' & v_0\pi^\dagger \\ v_3\pi^\dagger & -v_4\pi & v_0\pi & -u/2 \end{pmatrix} \otimes s_0, \tag{S2}$$

$$\mathcal{H}_{\rm SOC} = \begin{pmatrix} \xi\lambda_I^b s_z/2 & i\lambda_R^b s_-^\xi & 0 & 0 \\ -i\lambda_R^b s_+^\xi & \xi\lambda_I^b s_z/2 & 0 & 0 \\ 0 & 0 & \xi\lambda_I^t s_z/2 & i\lambda_R^t s_-^\xi \\ 0 & 0 & -i\lambda_R^t s_+^\xi & \xi\lambda_I^t s_z/2 \end{pmatrix}, \tag{S3}$$

where $\mathcal{H}_{\rm BLG}$ is the Hamiltonian of the BLG[S2] and $\mathcal{H}_{\rm SOC}$ is the spin–orbit coupling term describing the proximity induced Ising-type SOC with the parameters of λ_I^i and Rashba-type SOC parametrized with λ_R^i.[S3,S4] Here, s_i, with $i = \{0, x, y, z\}$, are the spin Pauli matrices and $s_\pm^\xi = \frac{1}{2}(s_x + i\xi s_y)$. In $\mathcal{H}_{\rm BLG}$, γ_i, with $i = \{0, 1, 3, 4\}$ describe the intra- and interlayer hoppings in BLG, as illustrated in Fig. S1.b, $v_i = \sqrt{3}a\gamma_i/2\hbar$ are effective velocities, with the lattice constant of the graphene $a = 2.46$ Å and Δ' is the dimer on-site energy. γ_0 is the nearest neighbour intralayer hopping, γ_1 is the interlayer hopping between the dimer sites, γ_3 describes the hopping between the non-dimer sites and γ_4 is the interlayer hopping between the dimer and non-dimer orbitals. In $\mathcal{H}$, $\pi = \hbar(\xi k_x + ik_y)$ and $\pi^\dagger = \hbar(\xi k_x - ik_y)$ are momentum operators measured from the K and K' valleys with the valley indices $\xi = \pm 1$. The parameter u is the interlayer potentials difference modelling the effect of an external electric field.

In our simulations we have used the following parameters: $\gamma_0 = 2.61$ eV, $\gamma_1 = 0.361$ eV, $\gamma_3 = 0.283$ eV, $\gamma_4 = 0.138$ eV and $\Delta' = 0.015$ eV.[S5]

In the main text, we show the spectrum near the K valley. Here, we show the difference between the K and K' valleys in Fig S2. The main difference, besides the opposite tilting

due to the trigonal warping, is the opposite spin polarization of the bands, which is the manifestation of the Kramers theorem, since the valley-Zeemann terms generate an opposite effective magnetic field in the two valleys. In the figures, we calculate the spin polarization as

$$\zeta_n = \sum_{X=A1,A2,B1,B2} |c_{X,\uparrow}|^2 - |c_{X,\downarrow}|^2. \tag{S4}$$

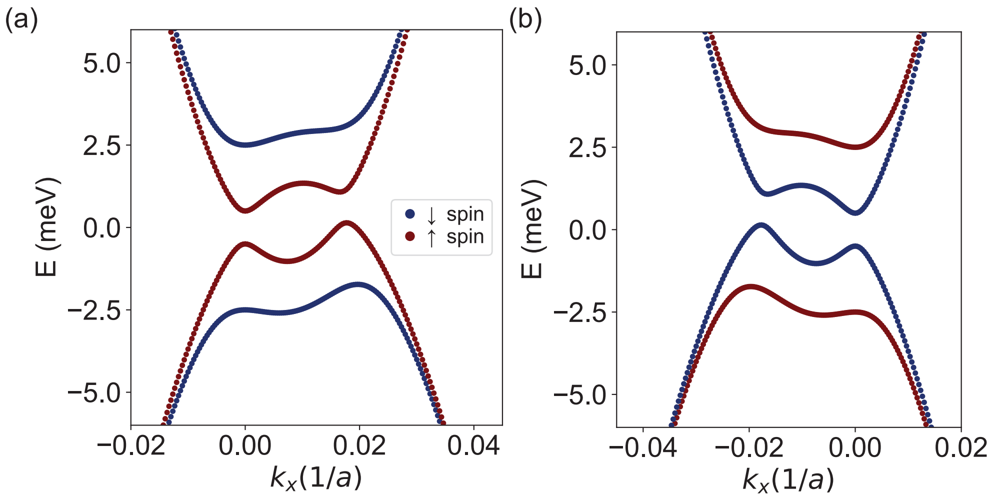

Figure S2: Calculated band structure using the parameters of $u = 3\,\text{meV}$ and $\lambda_I^b = -\lambda_I^t = 2\,\text{meV}$ (a) near the K valley and (b) near the K' valley.

Besides the spin polarization, the layer polarization α_n is also an important parameter of the model, which is defined as

$$\alpha_n = \sum_{s=\uparrow,\downarrow} |c_{A1,s}|^2 + |c_{B1,s}|^2 - |c_{A2,s}|^2 - |c_{B2,s}|^2. \tag{S5}$$

As shown in Fig. S3. at $u = 0$ the bands have no layer polarization, which can be lifted by increasing u. For $|u| > |\lambda_I^b|$, the low energy part of the conductance and valence bands becomes layer polarized with the opposite layer polarization of the valence and conduction bands.

For completeness, the band structure near the K-point is shown in Fig. S4. with $\lambda_I^b = \lambda_I^t$. In this case, the bands are spin split due to the SOC, which can be considered here as a Zeemann splitting in an effective magnetic field. Moreover, in this case, there is no gap at

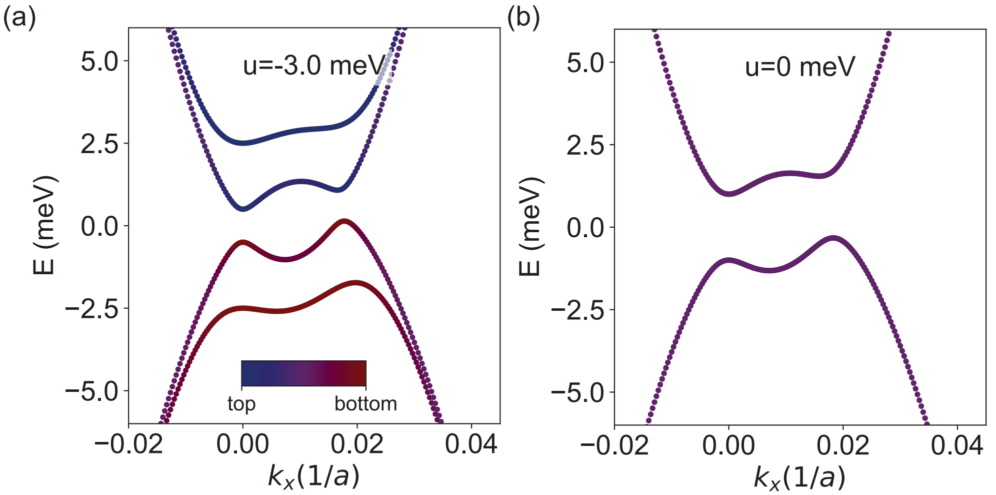

Figure S3: Calculated band structure using the parameter of $\lambda_I^b = -\lambda_I^t = 2\,\text{meV}$ (a) at $u = -3\,\text{meV}$ and (b) at $u = 0\,\text{meV}$. The color of the line corresponds to the layer polarization: the blue (red) points are fully polarized to the top (bottom) layer and the purple points are layer degenerated.

$u = 0$ and a gap only opens if $|u| > |\lambda_I^{b,t}|$.

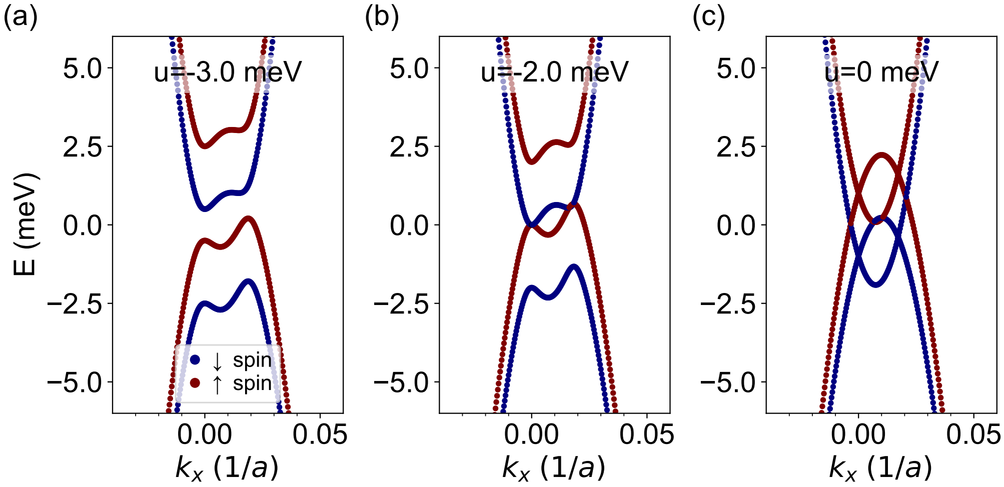

Figure S4: Calculated band structure using the parameter of $\lambda_I^b = \lambda_I^t = 2\,\text{meV}$ (a) at $u = -3\,\text{meV}$, (b) at $u = -2\,\text{meV}$ and (c) at $u = 0\,\text{meV}$. The color of the lines corresponds to the spin polarization.

S2.2 Landau level calculations

In high perpendicular magnetic field B, we replace the canonical impulse with the kinetic momentum $\hbar q_i = \hbar k_i - eA_i$, where A_i is the vector potential and introduce the magnetic

ladder operators as $\hat{a} = \sqrt{\frac{\hbar}{2eB}}\,(q_x + iq_y)$ and $\hat{a}^\dagger = \sqrt{\frac{\hbar}{2eB}}\,(q_x - iq_y)$, which satisfy $[\hat{a}, \hat{a}^\dagger] = 1$. In Eq. S2, we replace π and $\pi^\dagger$ with the ladder operators as $\pi = \sqrt{2eB\hbar}\hat{a}$ and $\pi^\dagger = \sqrt{2eB\hbar}\hat{a}^\dagger$ for the K valley and $\pi = -\sqrt{2eB\hbar}\hat{a}^\dagger$ and $\pi^\dagger = -\sqrt{2eB\hbar}\hat{a}$ for the K' valley. We neglect γ_3 in the Landau level (LL) calculations, which would introduce a small mixing of the zero LLs and the higher LLs. For the K valley the $\mathcal{H}_{\mathrm{BLG}}$ can be rewritten as

$$\mathcal{H}^{K}_{\mathrm{BLG}} = \begin{pmatrix} u & v_0\sqrt{2eB\hbar}\hat{a}^\dagger & -v_4\sqrt{2eB\hbar}\hat{a}^\dagger & 0 \\ v_0\sqrt{2eB\hbar}\hat{a} & u+\Delta' & \gamma_1 & -v_4\sqrt{2eB\hbar}\hat{a}^\dagger \\ -v_4\sqrt{2eB\hbar}\hat{a} & \gamma_1 & -u+\Delta' & v_0\sqrt{2eB\hbar}\hat{a}^\dagger \\ 0 & -v_4\sqrt{2eB\hbar}\hat{a} & v_0\sqrt{2eB\hbar}\hat{a} & -u \end{pmatrix} \otimes s_0, \tag{S6}$$

and for K' valley it is given by

$$\mathcal{H}^{K'}_{\mathrm{BLG}} = \begin{pmatrix} u & -v_0\sqrt{2eB\hbar}\hat{a} & v_4\sqrt{2eB\hbar}\hat{a} & 0 \\ -v_0\sqrt{2eB\hbar}\hat{a}^\dagger & u+\Delta' & \gamma_1 & v_4\sqrt{2eB\hbar}\hat{a} \\ v_4\sqrt{2eB\hbar}\hat{a}^\dagger & \gamma_1 & -u+\Delta' & -v_0\sqrt{2eB\hbar}\hat{a} \\ 0 & v_4\sqrt{2eB\hbar}\hat{a}^\dagger & -v_0\sqrt{2eB\hbar}\hat{a}^\dagger & -u \end{pmatrix} \otimes s_0. \tag{S7}$$

The full Hamiltonian in B field is also expanded with the Zeeman term $\mathcal{H}_Z = E_Z s_z$, where $E_Z = -\mu_B B$ is the Zeeman energy with the Bohr magneton μ_B. The eigenenergies E_{ξ,n,s_z} of $\mathcal{H} = \mathcal{H}_{\mathrm{BLG}} + \mathcal{H}_{\mathrm{SOC}} + \mathcal{H}_Z$ are defined as

$$\mathcal{H}\,|\xi, n, \sigma\rangle = E_{\xi,n,s_z}\,|\xi, n, \sigma\rangle\,, \tag{S8}$$

with the eigenstates of $|\xi, n, \sigma\rangle$. The ladder operators act on the Landau level wavefunctions as $\hat{a}\,|n\rangle = \sqrt{n}\,|n-1\rangle$ and $\hat{a}^\dagger\,|n+1\rangle = \sqrt{n+1}\,|n+1\rangle$. Following the footsteps of[S6], the same ansatz can be used to solve Eq. S8, which was used with induced SOC only in one graphene layer.

By solving the eigenvalue problem, the single-particle Landau level energies can be ob-

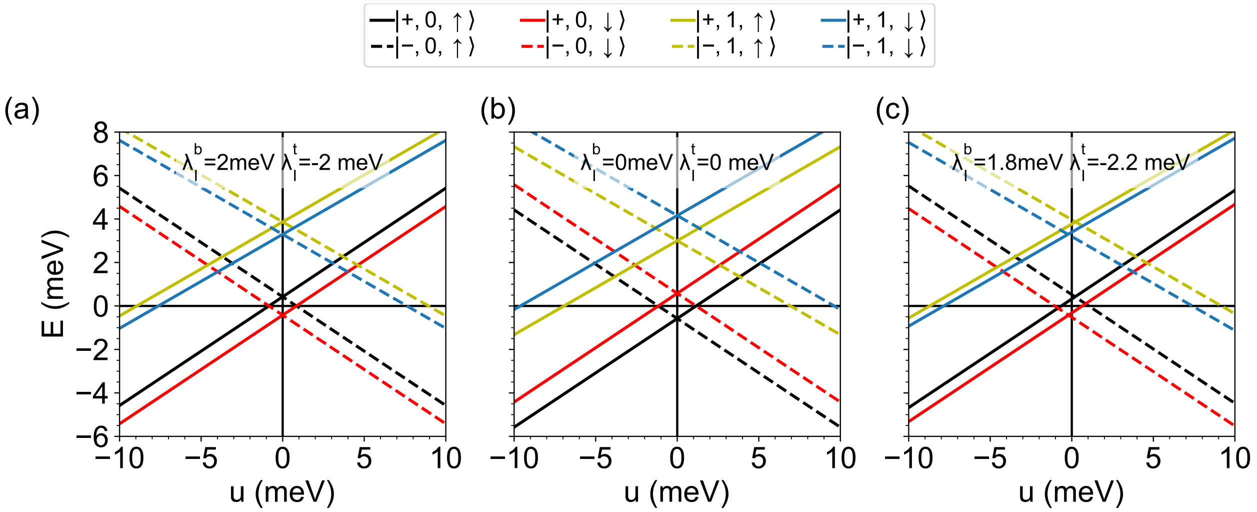

Figure S5: Single-particle zeroth LL spectrum as a function of u at $B = 10\,\mathrm{T}$ for (a) $\lambda_I^b = -\lambda_I^t = -2\,\mathrm{meV}$, (b) $\lambda_I^b = \lambda_I^t = -0$ and (c) $\lambda_I^b = 1.8\,\mathrm{meV}$ and $\lambda_I^t = -2.2\,\mathrm{meV}$.

tained. In Fig. 4a. in the main text, we plot the lowest 8 LLs with respect to u at $B = 8.5\,\mathrm{T}$. These LLs would be degenerate if we set every parameter to zero except γ_0 and γ_1, including the interlayer potential. In Fig. S5 we show the LLs in three different scenarios of λ_I: in panel (b) the LLs are shown if $\lambda_I = 0$. In this case, the LLs are spin split due to the Zeeman term and also split in the orbital index due to γ_0 and Δ'. A finite u further splits these LLs and their energy is linear in u. Since for the two lowest LL the spin and layer index becomes effectively the same, different valleys shift oppositely with a displacement field. By introducing a finite Ising-type SOC λ_I^b shifts the energies of the eigenstates of $|K, n, \sigma\rangle$ and λ_I^t shifts the energies of the eigenstates of $|K', n, \sigma\rangle$. Comparing panel (a) and (b), if $\lambda_I^t = -\lambda_I^b$, at high magnetic field, the spectrum seems very similar to the case without SOC, however, the order of the spin up and spin down levels flip. Moreover, the positions (u^*) where two LLs cross also change, which we defined as D^* crossings in the measurements in the main text. In the third case, shown in panel (c), when λ_I^t and λ_I^b have an opposite sign but their magnitudes are different, the u, $-u$ symmetry is lost leading to a non-zero $\pm u_3^*$ crossings.

In Fig. S6. and Fig. S7. we plot the Landau level crossings, with SOC and without SOC, for $\nu = 0$ and $\nu = \pm 1$, respectively. Without SOC the crossings go to zero as $B \to 0$ as opposed to the case of $\lambda_I^b = -\lambda_I^t \neq 0$. Comparing these figures with Fig 4. in the main text,

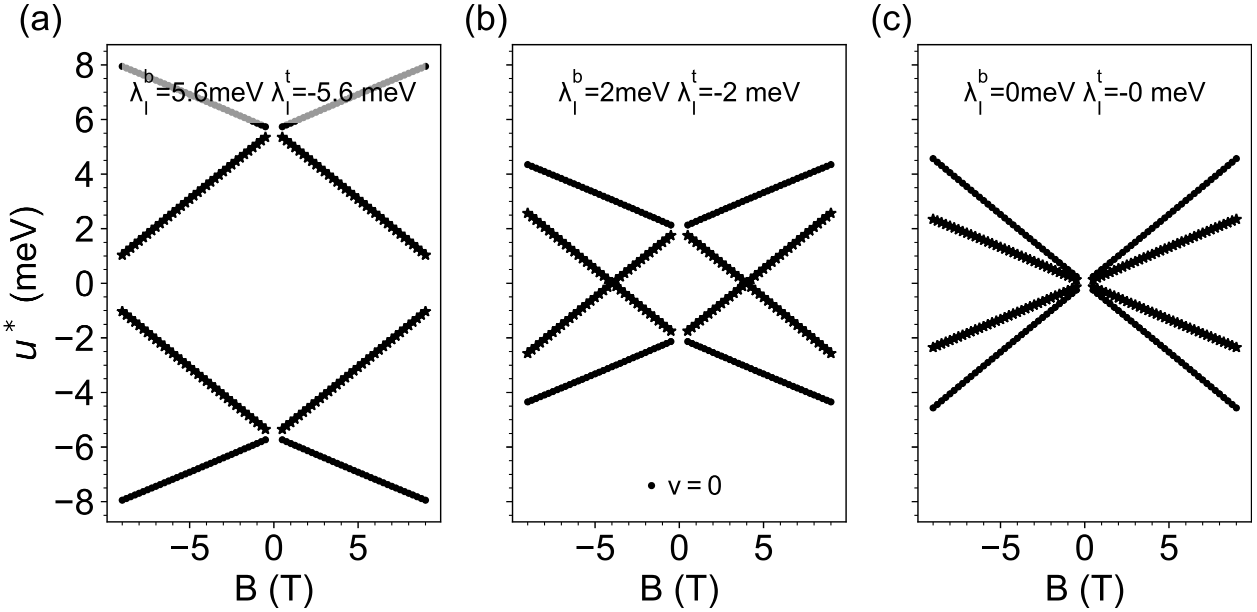

Figure S6: Landau level crossings at $\nu = 0$ as a function of B with (a) $\lambda_I^b = -\lambda_I^t = -5.6\,\mathrm{meV}$, (b) $\lambda_I^b = -\lambda_I^t = -2\,\mathrm{meV}$ and (c) $\lambda_I^b = \lambda_I^t = -0$.

the experiments show similarities to the model: the $\nu = 1$ crossings show similar tendency, so do the higher u_0^* branches in the $\nu = 0$ crossings. Likely the discrepancy between the model calculated in a single-particle picture and the experiment comes from the fact that in our calculations we neglect electron-electron interactions[S7].

S3 Determination of the lever arms

As discussed in the main text, to tune the charge carrier density n and the transverse displacement field D in the sample, gate voltages are applied to the metallic topgate (V_{TG}) and the graphite bottom gate (V_{BG}). The conversion from gate voltages to n and D is shown in Fig. S8 and is given by the following relation:

$$\begin{aligned} n &= \alpha_{TG} V_{TG} + \alpha_{BG} V_{BG} + n_0 \\ \frac{D}{\epsilon_0} &= \frac{e}{2\epsilon_0} \left(\alpha_{TG} V_{TG} - \alpha_{BG} V_{BG}\right) + \frac{D_0}{\epsilon_0}, \end{aligned} \tag{S9}$$

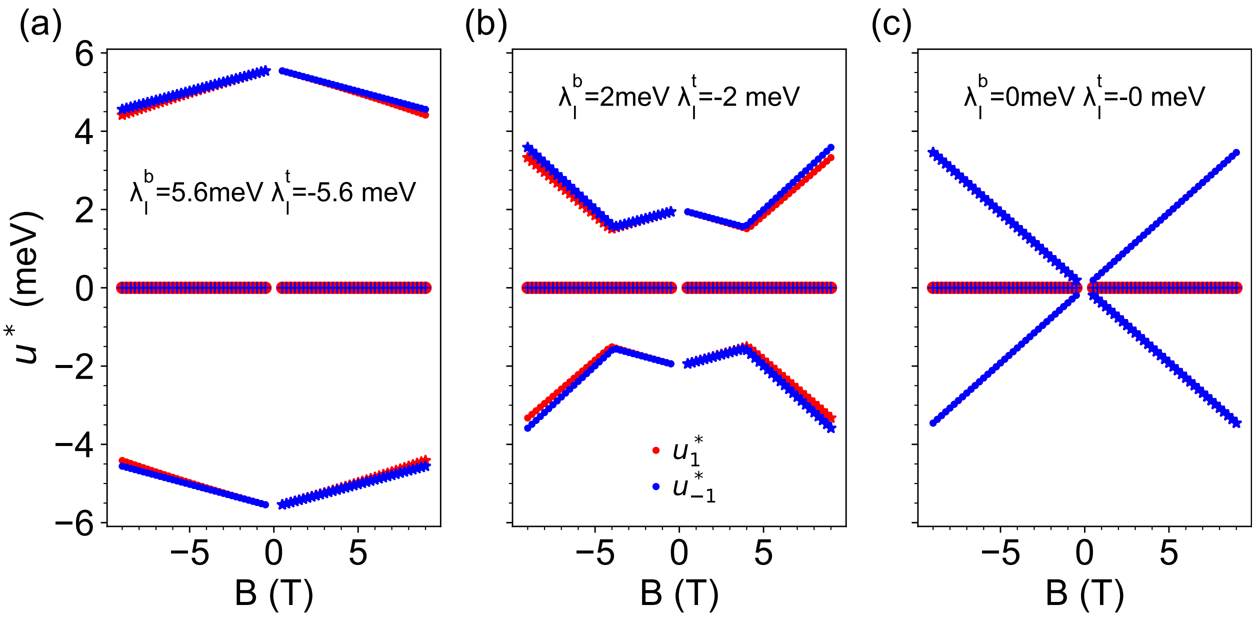

Figure S7: Landau level crossings at $\nu = \pm 1$ as a function of B with (a) $\lambda_I^b = -\lambda_I^t = -5.6\,\mathrm{meV}$, (b) $\lambda_I^b = -\lambda_I^t = -2\,\mathrm{meV}$ and with (c) $\lambda_I^b = \lambda_I^t = 0$.

where ϵ_0 is the vacuum permittivity, e is the elementary charge, $\alpha_{BG,TG}$ are the lever arms of the bottom and topgate, respectively, while n_0 and D_0 are the offset charge carrier density and displacement field. Since the lever arms are subject to change after the hydrostatic pressure is applied, originating from the compression of dielectrics, we determine them experimentally. First, the ratio of lever arms α_{BG}/α_{TG} can be obatined from gate voltage maps of the resistance (e.g. Fig. S8.a), as it is given by the slope of the charge neutrality line. Secondly, by measuring the fan diagram of Landau levels for $D = 0$, we determine the lever arms via the relation $\nu = nh/eB$ between the filling factor ν and the carrier density n for a given magnetic field B, where h is Planck's constant. The values of the lever arms can be found in Table S1.

p (GPa)	α_{BG} (10^{15} $\mathrm{V^{-1}m^{-2}}$)	α_{TG} (10^{15} $\mathrm{V^{-1}m^{-2}}$)
0	2.47 ± 0.08	2.81 ± 0.10
1.65	2.82 ± 0.07	3.12 ± 0.09

Table S1: Lever arms determined from quantum Hall measurements.

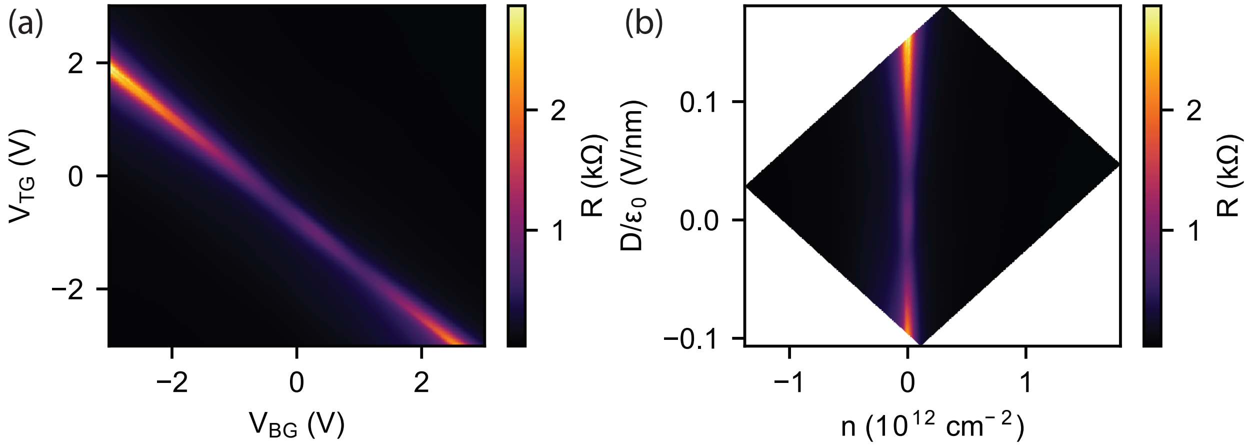

Figure S8: Conversion from a) gate voltages to b) charge carrier density n and transverse displacement field D.

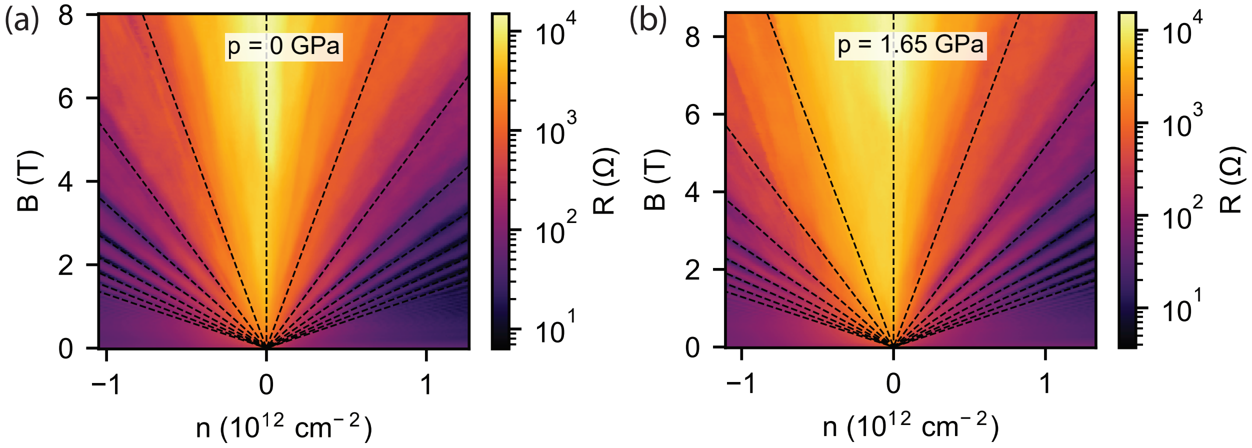

Figure S9: Landau fan daigram of the resistance for a) $p = 0$ and b) $p = 1.65\,\text{GPa}$ at $D = 0$. Dashed lines correspond to carrier densities with filling factors $\nu = 4k$, where $k \in \mathbb{Z}$.

S4 Extended activation data

Raw measurement data obtained while cooling the device is shown in Fig. S10 for $p = 0$ and $p = 1.65\,\mathrm{GPa}$. Similar maps were recorded while warming up the device from base temperature (not shown).

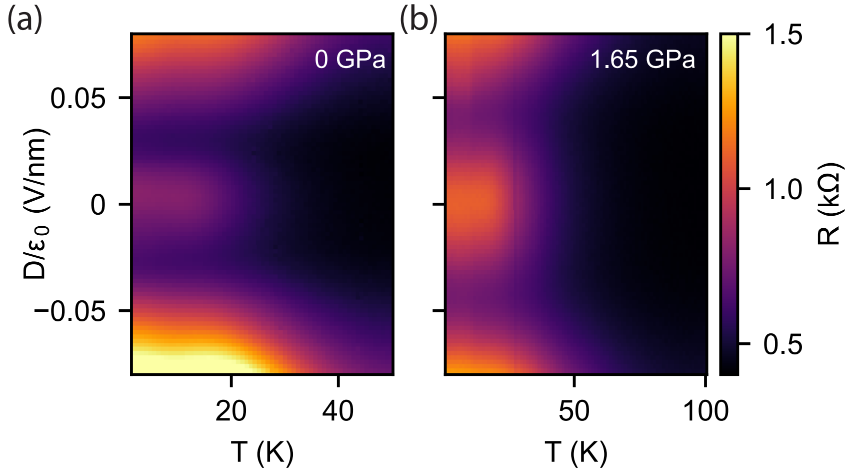

Figure S10: Temperature dependence of the resistance R as a function of D at $n = 0$ for a) $p = 0$ and b) $p = 1.65$ GPa.

S5 Determination of the SOI parameters

To obtain the SOI strength from thermal activation measurements, electric displacement field D has to be converted to the induced interlayer potential difference u. As described in the main text, this is done via the relation $u = -\frac{ed}{\epsilon_0 \epsilon_{\mathrm{BLG}}} D$. For ambient pressure, $d = 3.3\,\mathrm{Å}$ can be taken, similarly to pristine BLG. On the other hand, the value of ϵ_{BLG} available in the literature ranges from 2.6 [S8] to 6 .[S9] Since in the large u limit, the band gap induced by the displacement field is independent of the SOI parameters, by varying ϵ_{BLG}, we can effectively "fit" our model to the experimental data. Fig. S11.a shows the extended thermal activation data (partially presented in the main text) measured while warming up (red symbols) and cooling down (blue symbols) the sample for $p = 0$. As it is visible, in the band insulator regimes for $u \ll 0$ and $u \gg 0$, the data have different slopes. We take this effect into

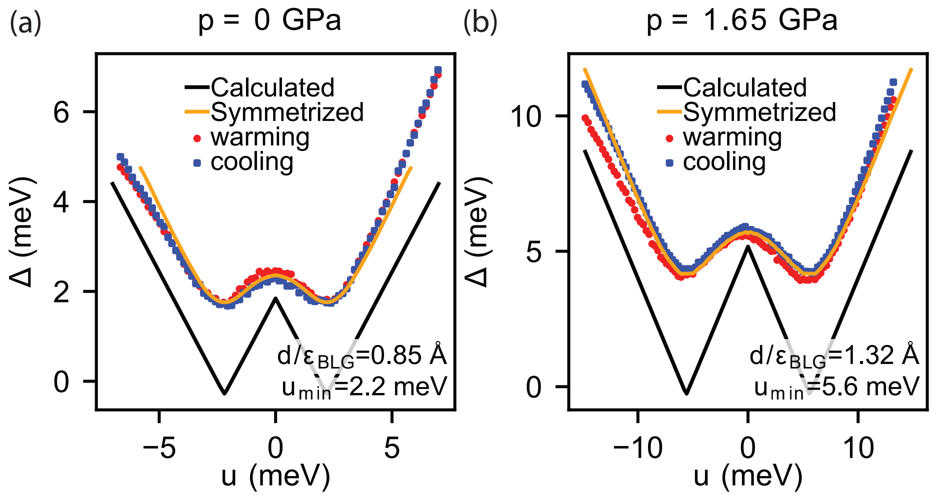

Figure S11: Band gaps determined from thermal activation measurements performed while warming up (red) and cooling down (blue) the device for a) $p = 0$ and b) $p = 1.65\,\text{GPa}$, respectively. Symmetrized curve with respect to $u = 0$ is shown in orange (see text for details) and the band gap calculated from the theoretical model is shown with the solid black line.

account by averaging of the two measurements and symmetrizing it with respect to $u = 0$ (solid orange line). In the next step, we determine ϵ_{BLG} by matching the slope of the high-u part of the data to match the slope of the theoretical model (solid black line). Finally, we numerically determine the position u_{min} of the band gap minimum of the symmetrized curve and take the Ising SOI parameters as $\lambda_t = -\lambda_b = u_{min}$. We estimate the lower and upper bound of the SOI parameter by fitting the slope of the theoretical curve to the $u \ll 0$ and $u \gg 0$ parts of averaged band gaps, respectively, yielding $\lambda_t = -\lambda_b = 2.2 \pm 0.4\,\text{meV}$ for $p = 0$.

In contrast to ambient pressure, at $p = 1.65\,\text{GPa}$, we expect both d and ϵ_{BLG} to change due to applied pressure. From theoretical predictions ,[S10,S11] we expect a change in the BLG interlayer distance of $\Delta d < 5\%/\text{GPa}$. However, to estimate the change in ϵ_{BLG} is more challenging. To be able to extract the SOI strength at $p = 1.65\,\text{GPa}$, we vary the d/ϵ_{BLG} ratio to match the experimental data to the model using the method described above. This way, for $p = 1.65\,\text{GPa}$, we extract $\lambda_t = -\lambda_b = 5.6 \pm 0.6\,\text{meV}$. The extracted increase in SOI strength is comparable to theoretical predictions using ab initio calculations.[S12] Furthermore,

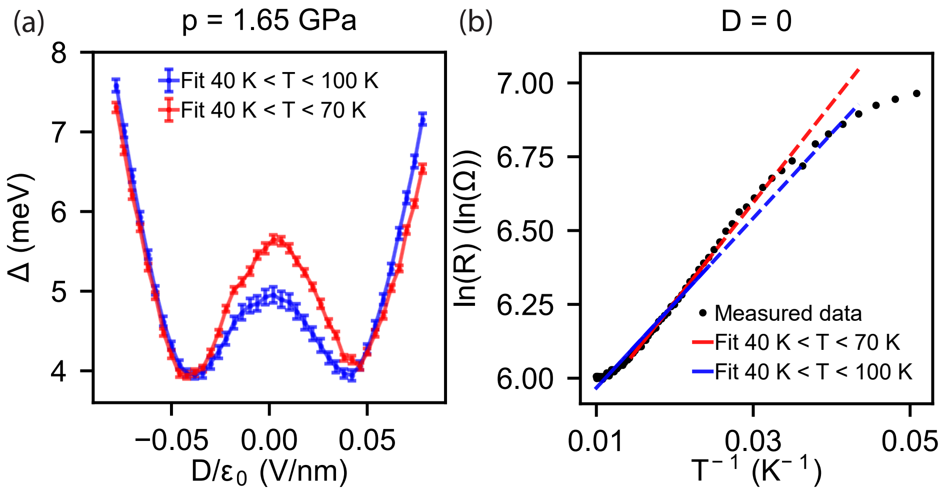

Figure S12: a) Band gaps determined from thermal activation measurements performed while cooling down dev S1 at $p = 1.65\,\mathrm{GPa}$, using a linear fit to the data according to the Arrhenius formula ($\ln R \propto \Delta/2k_BT$) in the temperature range $40\,\mathrm{K} < T < 70\,\mathrm{K}$ (blue) and $40\,\mathrm{K} < T < 100\,\mathrm{K}$ (red). Error bars represent the uncertainty of the fits. b) Examples for linear fits at $D = 0$ in the temperature range $40\,\mathrm{K} < T < 70\,\mathrm{K}$ (blue) and $40\,\mathrm{K} < T < 100\,\mathrm{K}$ (red) are shown with solid lines and are extended with dashed lines for better visibility. In this case, the difference in the slopes stems from the saturation of the resistance around 100 K.

the SOI parameters extracted from the minima give the same order of magnitude estimate as the extracted gaps at $u = 0$. Therefore it is clear, that the relative increase of the positions of band gap minima in D cannot alone stem from changes in the $d/\epsilon_{\mathrm{BLG}}$ ratio.

To estimate the robustness of our method, we extracted the band gaps from the thermal activation measurements at $p = 1.65\,\mathrm{GPa}$ using linear fits to different temperature ranges. These are shown in Fig. S12. for ranges of $40\,\mathrm{K} < T < 70\,\mathrm{K}$ (blue) and $40\,\mathrm{K} < T < 100\,\mathrm{K}$. The error bars on the figure show the error of the fit for given u and fixed temperature range. From this, we conclude that the uncertainty of the extracted band gaps is $< 20\%$. More importantly, the uncertainty of the slope of the $u \ll 0$ and $u \gg 0$ regimes and the positions of band gap minima is even less. Since in our analysis, these are the parameters that determine the extracted SOI strength, this results in an uncertainty of the $d/\epsilon_{\mathrm{BLG}}$ ratio and the SOI strength of $\sim 10\%$ that is comparable to the uncertainty estimated from the difference in the slopes of the $u \ll 0$ and $u \gg 0$ regimes.

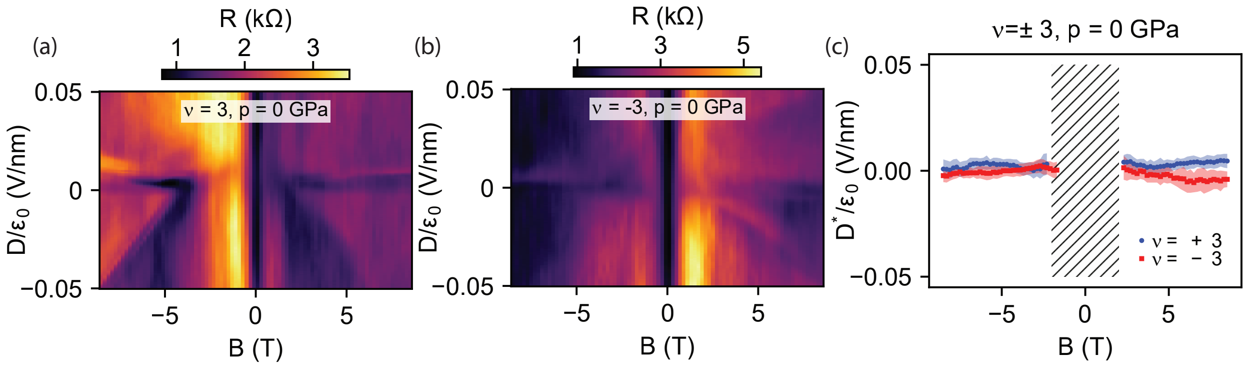

Figure S13: a,b) Measurements of LL crossings as a function of B for $\nu = +3$ and $\nu = -3$, respectively, for $p = 0$. c) Positions of the LL crossings extracted from the maps in a) and b) for $\nu = +3$ (blue) and $\nu = -3$ (red).

We also have to note that, although we assumed that $|\lambda_I^b| = |\lambda_I^t|$, our method of determining the SOI parameters is only sensitive to the absolute difference of the two parameters since this quantity defines the closing of the band gap. In other words, the minima of the $\Delta(u)$ functions shown in Fig. S11 are insensitive to a difference in the abolute values of $|\lambda_I^{b,t}|$ as long as $\left|\lambda_I^t - \lambda_I^b\right|/2$ is constant. However, we can estimate the asymmetry of $|\lambda_I^{b,t}|$ by measuring the $\nu = \pm 3$ LL crossings since, within our model, the positions of these crossings in u are separated by $u^*_{+3} - u^*_{-3} \approx |\lambda_I^t| - |\lambda_I^b|$, nearly insensitive to the magnetic field. Measurements of the LL crossings as a function of D and B for $\nu = +3$ and $\nu = -3$ are shown in Fig. S13.a,b, respectively. Fig. S13.c shows the extracted positions D^* of the crossings for $\nu = \pm 3$. As it is visible, for the most part of our magnetic field range, the positions of the crossings are indistinguishable. At higher magnetic field we see crossings at $u^*_{\pm 3} \neq 0$, indicating a small asymmetry of the SOC parameters. From these, we can estimate the upper bound of the asymmetry as $|\lambda_I^t| - |\lambda_I^b| < 0.4\,\text{meV}$ for $p = 0$.

S6 Extended quantum Hall measurement data

Additional data for the quantum Hall measurements at $p = 1.65\,\text{GPa}$ is shown in Fig. S14.

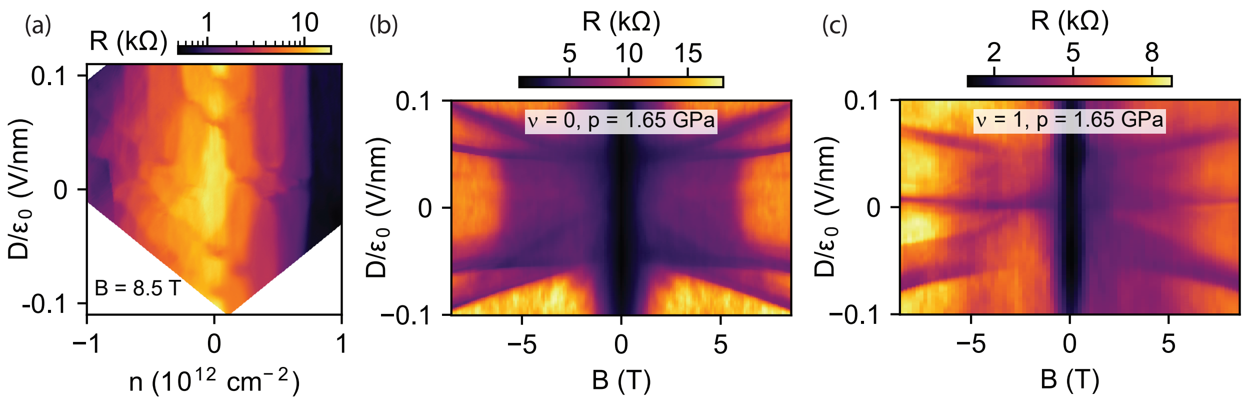

Figure S14: Extended data for Fig. 4. of the main text. a) n-D map of the resistance measured at $B = 8.5\,\mathrm{T}$ and $p = 1.65\,\mathrm{GPa}$. b,c) Measurements of LL crossings as a function of B for $\nu = 0$ and $\nu = 1$, respectively, at $p = 1.65\,\mathrm{GPa}$.

S7 Additional measurements on two-terminal devices

We performed additional measurements on two-terminal devices also shown in Fig. S1.a. Two different devices were measured between contacts E-F (dev S2) and F-G (dev S3) in separate pressurization and cool-down cycles. Two-terminal resistance maps of the devices as a function of n and D are shown in Fig. S15 at $p = 0$ and $p = 1.2\,\mathrm{GPa}$. These results are consistent with our observations on dev S1 in that the inverted phase is clearly visible and is enhanced by the applied pressure. Fig. S15.c and S15.f shows the comparison between line traces of the resistance measured at $n = 0$ as a function of D at $p = 0$ and $p = 1.2\,\mathrm{GPa}$ for dev S2 and S3, respectively. In both cases, the resistance at $D = 0$ is increased due to the applied pressure and the location of resistance minima (D^*) is increased by $\sim 25\%$.

We also performed thermal activation measurements on both devices. For reference, the raw measurement data is shown for dev S2 in Fig. S16.a for $p = 0$ obtained while cooling the sample from 100 K to base temperature. Due to the two-terminal geometry of these devices a large jump in resistance is visible that corresponds to the superconducting phase transition of the NbTiN contacts.

In order to extract the band gaps from these measurements additional data processing is required and the normal resistance of the contacts have to be subtracted from the high

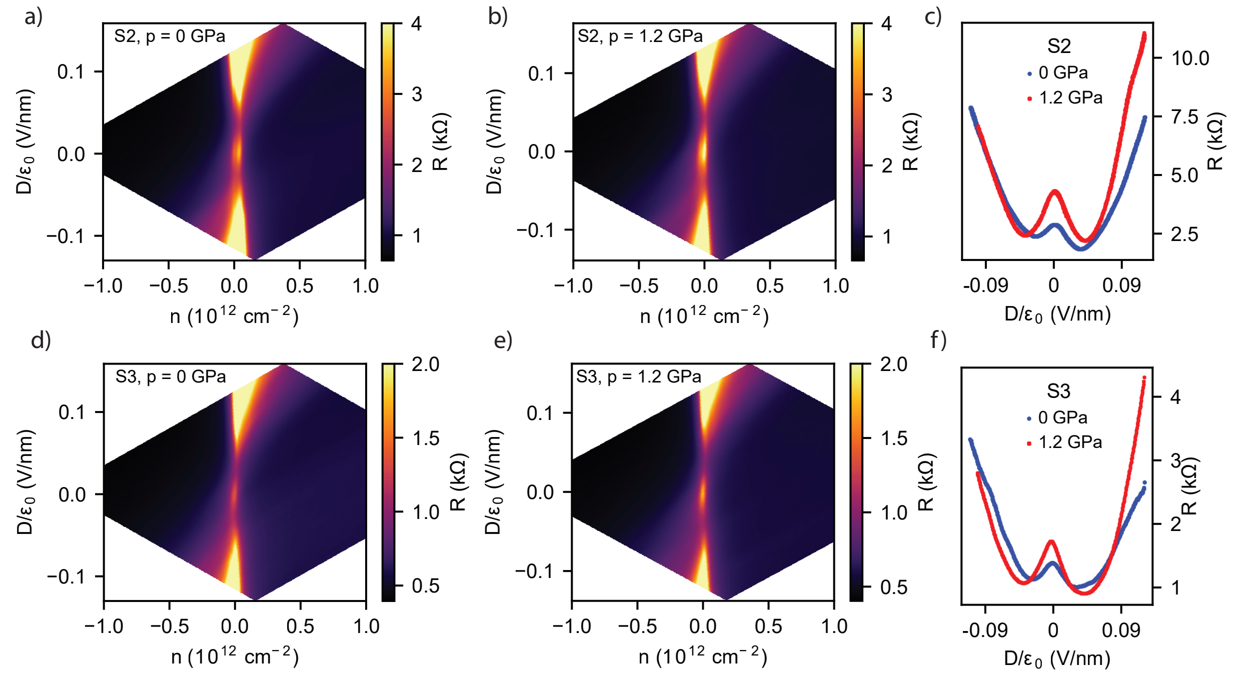

Figure S15: a,b) 2-terminal resistance map of dev S2 for a) ambient pressure and b) $p = 1.2\,\mathrm{GPa}$. c) Line traces of resistance for dev S2 as a function of D at $n = 0$ for ambient pressure (blue) and $p = 1.2\,\mathrm{GPa}$ (red). d,e,f) Similar resistance maps and comparison for dev S3.

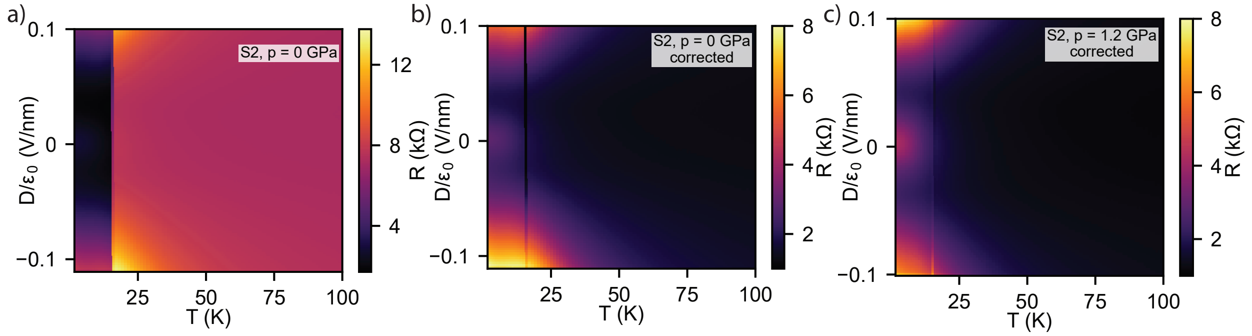

Figure S16: Temperature dependence of the resistance of device S2 as a function of D at $n = 0$ for a) $p = 0$. The jump in the resistance around 15 K corresponds to the superconducting phase transition of the NbTiN leads. Corrected data after the contact normal resistance was subtracted for b) $p = 0$ and c) $p = 1.2\,\mathrm{GPa}$.

temperature parts of the data. We subtract $R_{N,S2} = 5.90\,\mathrm{k\Omega}$ and $R_{N,S3} = 5.38\,\mathrm{k\Omega}$ for dev S2 and S3, respectively. The thermal activation data after the subtraction of the contact normal resistance is shown in Fig. S16.b and S16.c for dev S2 at $p = 0$ and $p = 1.2\,\mathrm{GPa}$, respectively. After the subtraction, a vertical line is visible that originates from the phase transition. We exclude these outliers from further analysis. Although this makes our data analysis less reliable, we can nevertheless extract the SOI strength using the same method as described in section S5. This yields $\lambda_t = -\lambda_b = 1.6 \pm 0.4\,\mathrm{meV}$ for $p = 0$ and $\lambda_t = -\lambda_b = 2.4 \pm 0.5\,\mathrm{meV}$ for $p = 1.2\,\mathrm{GPa}$ and $\lambda_t = -\lambda_b = 1.0 \pm 0.3\,\mathrm{meV}$ for $p = 0$ and $\lambda_t = -\lambda_b = 2.0 \pm 0.4\,\mathrm{meV}$ for $p = 1.2\,\mathrm{GPa}$ for dev S2 and S3, repsectively. These results also reproduce our findings for dev S1 as a significant increase in the extracted SOI strength is seen in all cases.

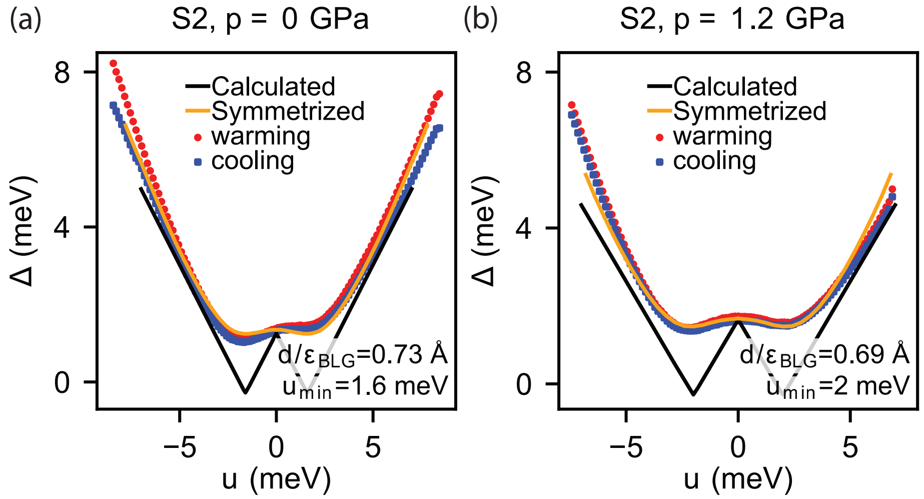

Figure S17: Band gaps determined from thermal activation measurements performed while warming up (red) and cooling down (blue) device S2 for a) $p = 0$ and b) $p = 1.2\,\mathrm{GPa}$, respectively. Symmetrized curve with respect to $u = 0$ is shown in orange and the band gap calculated from the theoretical model is shown with the solid black line.

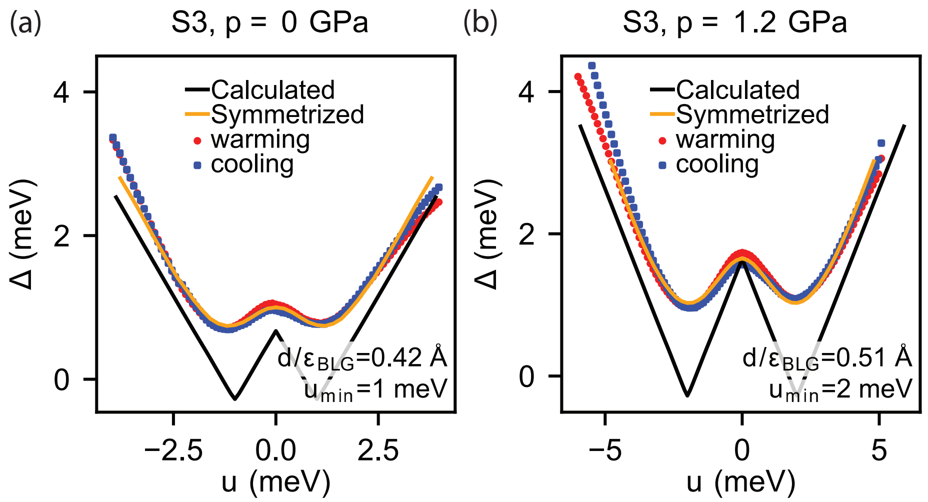

Figure S18: Band gaps determined from thermal activation measurements performed while warming up (red) and cooling down (blue) device S3 for a) $p = 0$ and b) $p = 1.2\,\mathrm{GPa}$, respectively. Symmetrized curve with respect to $u = 0$ is shown in orange and the band gap calculated from the theoretical model is shown with the solid black line.

S8 Additional data from Sample S4

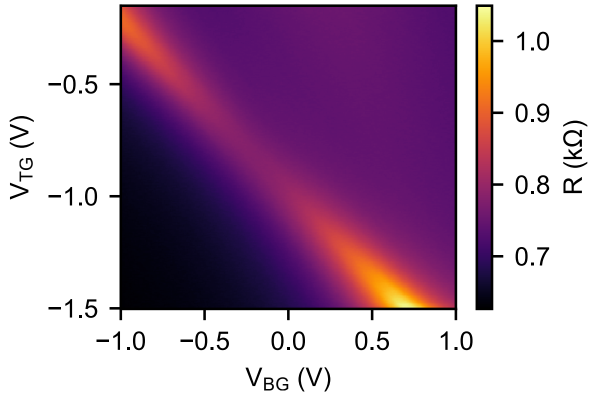

Figure S19: Differential resistance of a two-terminal device as a function of V_{BG} and V_{TG} that shows no signatures of band inversion.

As mentioned in section S1, not all samples showed signatures of band inversion. We attribute this to the lack of control over the rotation of WSe_2 layers. Based on theoretical predictions,[S13,S14] we argue that for certain rotation angles of the two WSe_2 layers (e.g. 0°), the sign of λ_I^b and λ_I^t can be the same which leads to the situation discussed section S2 and shown in Fig. S4 where no band gap is present at $D = 0$. For reference, in Fig. S19, we show

measurement data of a two-terminal device measured at 4 K, where no signatures of band inversion can be observed in the resistance map.

References

[S1] Fülöp, B.; Márffy, A.; Tóvári, E.; Kedves, M.; Zihlmann, S.; Indolese, D.; Kovács-Krausz, Z.; Watanabe, K.; Taniguchi, T.; Schönenberger, C.; Kézsmárki, I.; Makk, P.; Csonka, S. New method of transport measurements on van der Waals heterostructures under pressure. *Journal of Applied Physics* **2021**, *130*, 64303.

[S2] McCann, E.; Koshino, M. The electronic properties of bilayer graphene. *Reports on Progress in Physics* **2013**, *76*, 056503.

[S3] Konschuh, S.; Gmitra, M.; Kochan, D.; Fabian, J. Theory of spin-orbit coupling in bilayer graphene. *Phys. Rev. B* **2012**, *85*, 115423.

[S4] Zollner, K.; Fabian, J. Bilayer graphene encapsulated within monolayers of WS_2 or $Cr_2Ge_2Te_6$: Tunable proximity spin-orbit or exchange coupling. *Phys. Rev. B* **2021**, *104*, 075126.

[S5] Jung, J.; MacDonald, A. H. Accurate tight-binding models for the π bands of bilayer graphene. *Phys. Rev. B* **2014**, *89*, 035405.

[S6] Khoo, J. Y.; Levitov, L. Tunable quantum Hall edge conduction in bilayer graphene through spin-orbit interaction. *Physical Review B* **2018**, *98*, 115307.

[S7] Hunt, B. M.; Li, J. I. A.; Zibrov, A. A.; Wang, L.; Taniguchi, T.; Watanabe, K.; Hone, J.; Dean, C. R.; Zaletel, M.; Ashoori, R. C.; Young, A. F. Direct measurement of discrete valley and orbital quantum numbers in bilayer graphene. *Nature Communications* **2017**, *8*, 948.

[S8] Slizovskiy, S.; Garcia-Ruiz, A.; Berdyugin, A. I.; Xin, N.; Taniguchi, T.; Watanabe, K.; Geim, A. K.; Drummond, N. D.; Fal'ko, V. I. Out-of-Plane Dielectric Susceptibility of Graphene in Twistronic and Bernal Bilayers. *Nano Letters* **2021**, *21*, 6678–6683.

[S9] Bessler, R.; Duerig, U.; Koren, E. The dielectric constant of a bilayer graphene interface. *Nanoscale Advances* **2019**, *1*, 1702–1706.

[S10] Carr, S.; Fang, S.; Jarillo-Herrero, P.; Kaxiras, E. Pressure dependence of the magic twist angle in graphene superlattices. *Physical Review B* **2018**, *98*, 085144.

[S11] Yankowitz, M.; Jung, J.; Laksono, E.; Leconte, N.; Chittari, B. L.; Watanabe, K.; Taniguchi, T.; Adam, S.; Graf, D.; Dean, C. R. Dynamic band-structure tuning of graphene moiré superlattices with pressure. *Nature* **2018**, *557*, 404–408.

[S12] Fülöp, B.; Márffy, A.; Zihlmann, S.; Gmitra, M.; Tóvári, E.; Szentpéteri, B.; Kedves, M.; Watanabe, K.; Taniguchi, T.; Fabian, J.; Schönenberger, C.; Makk, P.; Csonka, S. Boosting proximity spin-orbit coupling in graphene/WSe2 heterostructures via hydrostatic pressure. *npj 2D Materials and Applications* **2021**, *5*, 82.

[S13] Péterfalvi, C. G.; David, A.; Rakyta, P.; Burkard, G.; Kormányos, A. Quantum interference tuning of spin-orbit coupling in twisted van der Waals trilayers. *Phys. Rev. Res.* **2022**, *4*, L022049.

[S14] Li, Y.; Koshino, M. Twist-angle dependence of the proximity spin-orbit coupling in graphene on transition-metal dichalcogenides. *Phys. Rev. B* **2019**, *99*, 075438.
